# Knowledge mobilization in the context of health technology assessment: an exploratory case study

**DOI:** 10.1186/1478-4505-10-10

**Published:** 2012-04-03

**Authors:** Monique F Fournier

**Affiliations:** 1 Institut national d'excellence en santé et en services sociaux (INESSS), 2021 rue Union, Montréal, QC H3A 2S9, Canada

**Keywords:** Knowledge mobilization, Transfer, Production, Dissemination, Implementation, Health technology assessment, Decision-makers

## Abstract

**Background:**

Finding measures to enhance the dissemination and implementation of their recommendations has become part of most health technology assessment (HTA) bodies' preoccupations. The Quebec government HTA organization in Canada observed that some of its projects relied on innovative practices in knowledge production and dissemination. A research was commissioned in order to identify what characterized these practices and to establish whether they could be systematized.

**Methods:**

An exploratory case study was conducted during summer and fall 2010 in the HTA agency in order to determine what made the specificity of its context, and to conceptualize an approach to knowledge production and dissemination that was adapted to the mandate and nature of this form of HTA organization. Six projects were selected. For each, the HTA report and complementary documents were analyzed, and semi-structured interviews were carried out. A narrative literature review of the most recent literature reviews of the principal knowledge into practice frameworks (2005-2010) and of articles describing such frameworks (2000-2010) was undertaken.

**Results and discussion:**

Our observations highlighted an inherent difficulty as regards applying the dominant knowledge translation models to HTA and clinical guidance practices. For the latter, the whole process starts with an evaluation question asked in a problematic situation for which an actionable answer is expected. The objective is to produce the evidence necessary to respond to the decision-maker's request. The practices we have analyzed revealed an approach to knowledge production and dissemination, which was multidimensional, organic, multidirectional, dynamic, and dependent on interactions with stakeholders. Thus, HTA could be considered as a knowledge mobilization process per se.

**Conclusions:**

HTA's purpose is to solve a problem by mobilizing the types of evidence required and the concerned actors, in order to support political, organizational or clinical decision-making. HTA relies on the mediation between contextual, colloquial and scientific evidence, as well as on interactions with stakeholders for recommendation making. Defining HTA as a knowledge mobilization process might contribute to consider the different orders of knowledge, the social, political and ethical dimensions, and the interactions with stakeholders, among the essential components required to respond to the preoccupations, needs and contexts of all actors concerned with the evaluation question's issues.

## Background

The last three decades have seen the number of health technology assessment (HTA) agencies and programmes expand and become part of many industrialized countries' healthcare systems [1-3]. HTA aims at informing healthcare policymakers, managers and practitioners of the "clinical consequences, but also the economic, ethical, and other social implications of the diffusion and use of a specific procedure or technique on medical practice" [4] (p. 431). Although a research on the impact of the NHS Health Technology Assessment Programme concluded that the latter had a high impact on policy and on practice [5], a systematic review of the impact of HTA on health policy showed that only 50 to 70% of HTA reports were used by decision-makers [3] (p. 9).

Finding measures to enhance the dissemination and implementation of their recommendations has become part of most HTA bodies' preoccupations [3,6]. In that context, the *Agence d'évaluation des technologies et des modes d'intervention en santé *(AETMIS), the Quebec government HTA organization, in Canada, engaged in a reflection on its strategies to improve the applicability and utilization of its recommendations.

Under the authority of the Health and Social Services minister, AETMIS was governed by a board of independent experts and provided advice and recommendations to the Department of Health and Social Services, as well as to other organizations and actors of the healthcare system. AETMIS has become the *Institut d'excellence en santé et en services sociaux *(INESSS) in January 2011.

At the time of our study, AETMIS' mandate was to evaluate the efficiency, safety, costs and cost-effectiveness, as well as the ethical, social, organizational and economic implications of health technologies and modes of intervention, in order to support decision-making. Its mission also consisted in conducting clinical guidance projects, such as the development of clinical guidelines in healthcare and social services.

When AETMIS observed that some of its projects had relied on innovative practices in knowledge production and dissemination, it commissioned a research in order to identify what characterized these practices and to establish whether they could be systematized.

During summer and fall of 2010, an exploratory case study of emerging practices at AETMIS was conducted in order to establish what determined the specificity of the latter's context of practices, and to identify the kind of knowledge the agency generated, how it was produced, by whom, for what purpose, and how it was disseminated and utilized. A literature review of models on how to transfer knowledge more effectively to decision-makers in the healthcare sector [7-12] was carried out to examine if they could apply to the agency's particular context, motivations and types of activities. The main objective of both the exploratory case study and the literature review was to conceptualize an approach to knowledge production and dissemination that was adapted to the mandate and nature of a HTA organization.

## Methods

A committee of researchers, project managers and communication officers was created to select six projects that were considered promising paths to follow in health technology assessment as well as in clinical guidance. Five were completed between 2005 and 2010, and one was ongoing. The objective was not to be representative of all of the Agency's practices. Each project was chosen because it reflected different aspects of emerging practices of knowledge production and dissemination for decision-making, either by its context and the issues it raised, or the types of evidence collected, the modes of interactions adopted, the moment the stakeholders got involved in the process, or by the way the results were disseminated. Three of them were related to health technology and clinical procedures assessment: 1) the use of class 3B and class 4 lasers and intense pulsed light sources for cosmetic procedures in non-medical settings [13]; 2) the introduction of advanced life support in emergency prehospital services in Quebec [14]; and 3) the chronic fatigue syndrome: state of the evidence and assessment of intervention modalities in Quebec [15]. Three projects were concerned with clinical guidance and support: 1) a systematic evaluation of ST-elevation myocardial infarction (STEMI) care [16,17]; 2) clinical guidelines for pediatrics obesity treatment [ongoing]; and 3) ministerial guidelines for mild traumatic brain injuries (MTBI) (2005-2010) [18]. Although social services became part of AETMIS' mandate in 2009, no project in this field was sufficiently developed at the time of our study to be examined.

An exploratory case study of these emerging practices appeared to be the most appropriate methodology. The aim was not to test a hypothesis, but to identify the main characteristics of the knowledge production and dissemination approach used in the selected projects. The reasons why innovative components were introduced in the process were documented and analyzed to unveil the rationality guiding the innovation as well as the specific nature of the context of practices.

For each project, the HTA reports and other complementary documents were analyzed, and semi-structured interviews with one of the researchers in charge of the project were carried out. The interviews were designed to collect information on the strategies used by the researchers to mobilize knowledge at each step of the process, that is, from the moment the agency received the request to the findings' dissemination and implementation. More specifically, we inquired as to whom they had consulted, at what phase, for what reasons, the nature of the interactions and the types of evidence collected, how the latter was utilized in the assessment, and how it guided knowledge production, dissemination and implementation. The use of two different methods (interviews and document analysis) for collecting data contributed to the validation of the analysis through a methodological triangulation [19]. The interviews were useful to document the unwritten process, the challenges of interacting with stakeholders, the use of informal consultations and the methodological choices related to the types of evidence needed. Taken together, the document analysis and the interviews offered a more accurate picture of the logic pertaining to the emerging practices. The selection of projects conducted by different persons and in different periods from 2005 to 2010 was also helpful to determine a tendency through time.

In order to compare our observations with the principal knowledge into practice frameworks, we undertook a narrative literature review of the most recent systematic literature reviews on the subject (2005-2010) and of articles describing such frameworks (2000-2010). Our intention was not to produce an exhaustive inventory of all the existing frameworks, but to get an overview of the ones which were the most referred to and considered dominant in the healthcare sector. We focused our attention on articles treating of the relationship between knowledge translation and healthcare decision making. We used three categories of key words: 1) Related to the knowledge into action process: knowledge translation or transfer, knowledge dissemination or diffusion, research utilization, use, uptake or implementation, knowledge brokering, knowledge exchange and sharing, diffusion of innovation, knowledge mobilization; 2) Related to decision making: policy making, public health, health policy, policy making; 3) Related to health, health care, social services, health technology assessment, clinical guidance or guidelines. We have essentially searched in the following databases: PubMed, EMBASE, Web of Science, Social Sciences Index, Social Services Abstracts, Social Work Abstracts, Sociological Abstracts and Repère. We have also consulted the grey literature, and used the snowball method (manual searches) for selecting the most frequently cited authors in the references. Documentary updates were performed until the date of article submission.

### Analytical framework

We have constructed our analytical framework on the basis of the elements that are taken into consideration by most knowledge transfer or translation models. Most models share a similar starting point, that is, how to improve the use of knowledge by decision-makers and practitioners [10,11,20-22]. In that perspective, the type of knowledge that needs to be transferred into practice mainly concerns research results [20], whereas other types of knowledge related to the context of application and practice are analyzed to identify the barriers and facilitators to the uptake of research findings [8,21,23,24]. Interactions between producers and users are deemed essential in a number of models to understand and adapt the dissemination and implementation strategies to the context of utilization [10,21,24]. How the nature of these interactions is described in the models depends on the conception of their role in the process of knowledge production and its utilization. That is why, as we will see in more details in the discussion section, the nature of the interactions may either be unidirectional, bidirectional or multidirectional.

For each project of our exploratory case study, the following dimensions were examined and compared: as the starting point, the request's origin and context, the ensuing evaluation question and its related issues; the types of evidence needed and the data collection methods used; the involvement of stakeholders, the moment and nature of interactions; finally, the dissemination strategies and the implementation outcomes when available.

## Results: observations and discussion

The following section intends to draw the most significant elements from the different projects in order to offer a portrait of the process of knowledge production, dissemination and implementation in a HTA agency such as AETMIS. We will analyze in a general manner the request's origin and context, the types of evidence required to address the issues related to the question of evaluation, and the role of stakeholders in the whole process. The main characteristics of each project are presented in Table 1.

**Table 1:** Characteristics of cases studied

	Request's origin	Evaluation question	Types of issues	Types of evidence	Key stakeholders	Types of interaction	Dissemination strategies
**CFS**	Department of Health and Social services (DOHSS) responding to pressures from a patient association	-Synthesis of evidence on CFS and its treatments -Patients' needs -Actual modes of intervention -Degree of professional education and training on CFS	-Patients needs, values and preferences -Professional -Organizational	-Scientific -Scientific on context -Colloquial	-DOHSS -Patient association -CFS patients -Health and social services professionals and their supporting organizations -Public health insurance agency	-Inquiry (semi-structured interviews) -Consultations: individual and group interviews, online survey, etc	-Report sent to stakeholders -Document of sensitization for the public sent to patients' network and through the network of health and social services -Article published in a medical journal

**Laser 3B, 4 and IPL**	DOHSS responding to concerns towards risks raised by an association of dermatologists	Risks related to the use of laser class 3B and 4 and IPL for cosmetic procedures by non-physicians, without medical supervision	-Safety -Regulatory and legal frame -Professional (training)	-Scientific (safety) -Scientific on context -Colloquial evidence	-DOHSS -Association of dermatologists -Association of aestheticians -College of physicians -Sectoral committee on personal services workers -Concerned government departments (employment, education, health and social services)	-Collaboration and knowledge sharing and exchange	-Interactions with key actors through the whole process, including discussions on recommendations prior to publication -Report sent to stakeholders -Conferences

**Advanced life support (ALS) in emergency prehospital services**	DOHSS responding to a political crisis over pressures from an emergency services organization to introduce ALS	-Role of ALS in emergency prehospital services -Efficacy and conditions of implementation	-Efficacy and effectiveness -Organizational -Professional (training)	-Scientific (efficacy and safety) -Scientific on context -Colloquial	-DOHSS -Emergency services organisation (managers, physicians, paramedics) -DOHSS (emergency medical services) -College of physicians	-Consultation	-Widespread diffusion due to a highly mediatized and politicized context of publication and consensus of all parties around the recommendations

**Mild traumatic brain injuries (MTBI) **	DOHSS due to discrepancies in the interventions on MTBI	-Determine clinical and organisational parameters to guide interventions for MTBI	-Clinical practices -Organization of care and services	-Scientific -Scientific on context -Colloquial	-DOHSS -Public automobile insurance agency -Associations of health centers -Concerned medical associations -College of physicians -regional representatives -traumatology network	Collaboration and empowerment	-From the beginning through a stakeholders committee -Material sent to all concerned centers (guidelines, DVD, emergency posters, brochures, flyers) -Training offered -Monitoring process

**STEMI care**	DOHSS in response to time delays to treatment for STEMI patients	-Systematic field evaluation of STEMI care (primary study)	-Organizational -Clinical	-Scientific: Primary data on time delays to treatment	-DOHSS -Paramedics, emergency physicians, cardiologists, hospital managers, regional agencies managers -Tertiary cardiology expert committee -Regional health agencies -Health centers (professional services, emergency and cardiology services) and teaching hospitals -Emergency services organizations	-Information -Presentation of the project to stakeholders throughout the province -Training of hospital personnel in data collecting	-Presenting results in person to stakeholders throughout the province -Results on CD sent to all hospital professional services -Publication in a medical journal -Conferences -Collaborations with other provinces -Implementing of a monitoring mechanism by planning the conduct of a STEMI two years later

**Clinical Guidelines For Paediatric Obesity treatment**	Department of Health in response to the increasing prevalence of paediatric obesity and absence of clinical guidelines for its treatment	Clinical guidelines on paediatric obesity treatment	-Support clinical practice -Health care and services organisation -Patients' values and preferences	-Scientific (safety, efficacy of treatments) -Scientific on context -Colloquial	-Department of Health and Social services -Concerned health and social services professionals (primary, secondary, tertiary healthcare settings) -Parents -Professional orders	Collaboration throughout the process through multiple committees for the determining of clinical issues, appraisal of scientific evidence, formulation of recommendations, advices on guidelines format	To be determined with the stakeholders, who will collaborate to the guidelines' dissemination

### Projects' starting point

As all projects at AETMIS, each of the ones studied began with a request presented to the Agency, in response to a problematic situation that calls for a decision to be made, and eventually an action to be taken. In the cases we have analyzed, the situations leading to a request were as diverse as a political crisis, concerns expressed by an interest group regarding the risks inherent in the use of a certain type of medical device, patients pressing the Minister of Health and Social Services to put forward specific care and services, the absence of clinical guidelines for a particular medical condition, or the need to decrease time delays to treatment in order to reduce morbidity and mortality.

In general, when AETMIS receives a request, exchanges with the decision-maker and sometimes with other actors are required to circumscribe the problem's context and determine the issues it raises. These may be of a technological, political, organizational, social, professional, ethical, economic or juridical nature. During this phase, AETMIS and the initial decision-maker agree on the dimensions that will be included in the evaluation question and on those to be excluded, as well as on the project's format and objectives.

For example, in the projects under study, the objectives were either to define clinical practice guidelines and protocols to support primary-care management of a specific illness, to identify the needs in professional training or in the organization of patient care and services, to evaluate the efficiency and conditions under which a new type of procedure should be introduced, or to establish what kind of regulation should be put forward by decision-makers to minimize the risks related to the use of a medical technology. In all cases, the purpose of the advice, recommendations or clinical guidelines were to inform decision-makers as to the actions to be undertaken in order to find solutions as regards the initial problematic situation, whether it be at the level of health policies, the organization of healthcare and services or in clinical practices.

### Types of evidence

Most projects have used the three types of evidence as distinguished by Lomas et al. [25]: 1) scientific evidence, defined as "knowledge that is explicit (codified and propositional), systemic (uses transparent and explicit methods for codifying), and replicable (using the same methods with the same samples will lead to the same results)"; 2) scientific evidence on context, which is "evidence about attitudes, implementation, organizational capacity, forecasting, economics/finance, and ethics"; and 3) colloquial evidence, which encompasses "evidence about resources, expert and professional opinion, political judgment, values, habits and traditions, lobbyists and pressure groups, and the particular pragmatics and contingencies of the situation" (p. 1-3).

The question of evaluation and its related issues determined the types of evidence required and how the evidence was to be collected and interpreted for conclusions and/or recommendations. Consequently, the methodology used to document the issues varied according to their nature. As illustrated in Table 2, the project on CFS relied on systematic reviews, grey literature reviews, primary qualitative research, interviews with groups and individuals, an online survey, etc. In comparison, the development of the guidelines for pediatric obesity required systematic reviews, expert opinions, group discussions and forums with intended users, patient representatives and partners, etc.

**Table 2:** Chronic Fatigue Syndrome: Evidence needed in relation to evaluation objectives

	Evaluation question	Type of evidence	Data collection strategies
**Objective 1**	1) To review the state of evidence concerning CFS and best practices for its management	Scientific evidence	-Systematic review of studies on the efficacy and safety of CFS treatments -Review of the clinical practice guidelines for the diagnostic and therapeutic management and rehabilitation of CFS patients

**Objective 2**	2) To identify the patients' needs in the area of healthcare and services	Scientific evidence Scientific evidence on context	-Systematic review of the literature -Review of clinical practice guidelines -Primary qualitative study based on semi-structured interviews with CFS patients

**Objective 3**	3) Determine the education/training needs of the health professionals involved in their application	Colloquial evidence on attitudes, opinions and experience of CFS	-Individual and group interviews -Online survey -Consultations with over 40 care providers and key stakeholders, etc

**Objective 4**	4) Assess the intervention modalities in Quebec, as well as organizational issues and its associated costs	Scientific evidence on context	-Review of services offered in several European countries and other countries, such as Australia -No available data on the prevalence of this illness in Quebec

The assessment of the CFS revealed the health professionals' lack of knowledge about the syndrome, their skepticism about its actual existence, their lack of experience in applying the therapies recognized as effective, and the absence of structured care or services for patients suffering from this illness. The ensuing recommendations were primarily determined in relation to the evidence concerning the patients' need for their syndrome to be recognized as a genuine illness in order for care and services to be developed and proposed. Thus, recommendations mainly focused on professional training and organizational measures [15].

Most cases we have studied demonstrated that scientific evidence based on systematic reviews of randomized controlled clinical trials, although mandatory, was rarely sufficient for recommendation making. In fact, in two cases, evidence from clinical findings proved insufficient or missing; thus reports had to ground most of their recommendations on scientific evidence on context and/or on colloquial evidence, which were validated through a rigorous triangulation of the collected data.

For example, in one of the projects under study, the evaluation question was to assess the risks related to the use of class 3B and class 4 lasers and intense pulsed light sources for cosmetic procedures by operators other than physicians or health professionals, without medical supervision [13]. The scientific evidence showed that although there were "adverse effects, minor and transient for the most part, in some cases serious," there were insufficient scientific evidence to determine the frequency and severity of these effects and no scientific study on the relation between adverse effects and the types of professionals using them.

The contextual evidence however revealed a gap in the legal and regulatory provisions framing the use of lasers and IPL by non-physician operators.

Thus, given the potentially serious adverse effects and the possibility of interference with the field of medicine, the ensuing recommendations were for competent authorities:

-to establish which cosmetic procedures could be performed by non-physicians and which ones not;

-to subject those not considered under the Medical Act to minimum quality-assurance measures;

-to standardize the required occupational skills;

-to develop vocational training and qualification programs as a means of determining occupational eligibility requirements; and

-to inform the public of the risks inherent in these technologies.

### The stakeholders

The interactions with stakeholders have been recognized as helpful in identifying the needs, interests and values of patients, practitioners and decision-makers, and understanding the professional, organizational, social and cultural contexts in which they evolve. Varvasovsky and Brugha [26] (p. 341) define stakeholders as "actors who have an interest in the issue under consideration, who are affected by the issue, or who - because of their position - have or could have an active or passive influence on the decision-making and implementation processes. They can include individuals, organizations, different individuals within an organization, and networks of individuals and/or organizations, i.e. alliance groups." Moreover, stakeholders are actors who possess knowledge, whether of an expert or experiential nature. Thus, they are able to bring new light and understanding to the problem area under study and contribute to refining it throughout the process.

In the majority of the projects we have analyzed, most key stakeholders concerned with the issues raised by the question of evaluation were represented in the process, particularly the individuals or organizations that were accountable and responsible for implementing the recommendations. In one case, although all key stakeholders had been identified and efforts were made to inform them of the ongoing assessment, one of the interest groups was overrepresented in the process. Since that particular group was reluctant to accept the results, AETMIS had to diversify its strategies of dissemination. This particular experience showed that increased interactions with the various key stakeholders could contribute to preventing one of the interest groups from monopolizing and defining the issues. Of course, this would involve taking into account the power struggles between them, their interests and the role they play with respect to the problem under study, as well as the personal and professional implications of the implementation of certain measures. Researchers from AETMIS and authors such as Thompson et al. [27] have also noted the importance of consulting stakeholders who are recognized as leaders by their peers.

Interactions not only lead to relevant information that cannot always be found in the literature (it may not have been published, or it may exist in for-internal-use-only documents, etc.), but also to other stakeholders who may be concerned and knowledgeable about the subject. One interviewed AETMIS researcher talked about a snowball approach, where one stakeholder led to another, and so on, until all the pieces of the puzzle were put together.

The degree of interaction with the stakeholders depended on the issues under examination, the nature of the actions these entail and the type of evidence necessary for assessment. We have observed all five degrees of stakeholder participation in the evaluation, as described by Patton [28]: information, consulting, involvement, collaboration and empowerment (p. 80-1). For example, parents and healthcare professionals have been collaborating since the beginning in the development of the clinical guidelines for pediatric obesity treatment. They have discussed clinical questions and even designed one and will validate the recommendations. Experts and other actors (professional orders, associations, etc.) have also been consulted throughout the process. The collaboration of stakeholders is considered a criterion of quality of clinical guidelines by the Appraisal of Guidelines Research and Evaluation (AGREE, http://www.agreetrust.org/) instrument. And according to Sorenson et al. [3], it constitutes an essential condition for their appropriation and for the utilization of knowledge in HTA as well. In one of the projects in HTA and two in clinical guidance that we have observed, there was literally a co-construction of knowledge by the key stakeholders. In the two cases where the recommendations were published, the stakeholders were expecting them and ready to engage in the actions proposed. They then naturally took the lead in passing the information to their peers.

### Dissemination and implementation

The dissemination and implementation of the recommendations seemed to vary according not only to the degree and purpose of the interactions with the key stakeholders, but also to the criteria by which they were selected. Thus, as previously mentioned, if key stakeholders were ignored or underrepresented in the process, it would seem that more efforts were required in order to disseminate the results at the end of the process.

We have observed other factors that had an impact on the dissemination and implementation of recommendations, including the following:

- The context generating the request and the issues that were addressed. One of the projects was assessed in a context of a highly publicized political crisis which had fostered expectations among the key stakeholders. Not only did they contribute by providing expertise, information and data, but they also awaited the report's conclusions with great interest. Since the report was concluded in a very short time, their interest was maintained.

- The need to fill a gap that was already acknowledged by the stakeholders. In one case, stakeholders were already engaged in a problem-solving process before the project started. AETMIS joined forces with them and helped come up with an integrated and concerted solution. At the end of the project, a meeting with all the stakeholders was organized to discuss how they would apply the recommendations.

- The inclusion of a monitoring process. Two of the projects have led to the implementation of a form of monitoring since their publication. In one case, the project was designed in such a way that a second evaluation could be planned to measure, two years after the first study, the progress with time delays to treatment. In the other case, ongoing cooperation mechanisms with all the key stakeholders of a network of services for a specific health condition were put forward in order to exchange new evidence and decisional algorithms. The efficiency of this network relies on a tight and constant follow-up of the implementation of measures and adjustments. A Website was created to monitor the activities and performance of services, to provide access to articles and references in the field, and to publish the validated protocols in the network. For each data update, posters of decisional algorithms were transmitted to all concerned centers and regional agencies.

- The use of diverse dissemination strategies. A variety of communication tools and dissemination strategies is sometimes used in order to multiply the entry points to different stakeholders. For example, in one case, the results were presented, in person, to all concerned stakeholders. A CD containing the results was sent to the hospitals' professional services. A scientific article was published in a medical journal [29], which gave great credibility to the results. The team has collaborated with renowned experts and leaders throughout Canada and participated in many conferences.

## Discussion

In light of these observations, what can we learn about the knowledge production, dissemination and implementation process in a HTA context? Although HTA organizations often refer to the dominant knowledge translation models to define their practices, our observations reveal an inherent difficulty as regards applying such models to HTA and clinical guidance practices.

In this section, we will discuss what distinguishes the practices we have analyzed from the fundamental elements of the dominant knowledge translation models. Then, we will explore another approach that might be more adapted to the specific context of HTA and clinical guidance.

### Different contexts, different premises

The principal knowledge translation models in the healthcare field have been designed in a research context. The use of knowledge in decision-making constitutes one of the main priorities of the health sector's research organizations and funding bodies. These bodies deplore that findings from clinical research are insufficiently applied in practice. Thus, patients may not receive proper care, resulting in mortality, morbidity and impacts on their quality of life, as well as greater expenditures for healthcare systems [11,20,21,30].

Many authors insist on the necessity of bridging the gap between research findings in healthcare and their utilization by decision-makers, including patients, practitioners, managers and policy-makers. This is why most models concerned with the uptake of research findings consist of "methods for closing the gaps from knowledge to practice" [7] or facilitate the application of research products and synthesis. Sharon Straus, Jacqueline Tetroe and Ian Graham, prominent authors affiliated with the Canadian Institutes of Health Research, use the concepts of knowledge translation and knowledge to action interchangeably to refer to these methods [24]. Whether these are called knowledge translation, knowledge transfer or exchange, knowledge uptake, research utilization or implementation science, knowledge dissemination or diffusion, the problem of moving evidence into practice generally constitutes the premise underlying their conceptualization [7,10,11,31,32].

For a HTA organization such as AETMIS, the whole process starts with a question of evaluation asked in a problematic situation for which an actionable answer is expected. Thus, the objective is not to move evidence into practice, but to produce the evidence necessary to respond to the decision-maker's request. The mandate to support decision-making and clinical practice, with actionable recommendations and guidelines, has implications as regards the definition and production of knowledge, as well as concerning the representation of action and the role of stakeholders in the process.

### What kind of knowledge?

In the most common knowledge-to-action frameworks, knowledge primarily means research findings, more particularly scientific evidence from systematic reviews, meta-analysis and controlled randomized trials [20].

From that perspective, contextual and colloquial evidence are considered useful in adapting research findings to the context of their application [10,25] or identifying the barriers and facilitators that may have an impact on the utilization of research findings [7,20,21]. Thus, knowledge management (e.g. timeliness and relevance, personal contact between researchers and policy makers, inclusion of opinion leaders or knowledge brokers in research planning, etc.), organizational and structural characteristics (e.g. facilities, resources, financial incentives, etc.), as well as personal and professional characteristics (e.g. skills, attitudes, experiences, tacit knowledge, clinical judgment, etc.) will be interpreted as facilitators for or barriers to knowledge translation [8,21]. For that matter, Straus et al. [7] have identified over 250 barriers.

Thus, there is a tendency to consider, on the one hand, evidence from research that needs to be disseminated and applied, and on the other, knowledge from the implementation context that influences, positively or otherwise, the practitioners or decision-makers to use research findings. Such a tendency presupposes the existence of a hierarchy as regards the different types of evidence.

This hierarchization of evidence can also be found in HTA, where some authors will categorize timeliness, financial constraints, lack of transparency, and poor professional training, for example, as factors affecting the uptake of HTA findings [3,6]. However, there are other authors in HTA and evaluation who will regard contextual and colloquial evidence more as complementary for establishing the effectiveness, appropriateness, feasibility and acceptability of a technology or procedure. For these authors, the knowledge-to-action process will be guided by the following interrelated questions: Can it work (efficacy)? Can it work here (effectiveness)? Should we do it here (appropriateness)? How should we do it here (implementation) [33,34]?

Nonetheless, the latter approach to evidence still appears too restrictive to describe the HTA practices we have observed. When, for example, the question of evaluation concerns guidelines or patients' needs, the enquiry will not begin with "Can it work?", but with "What are the different types of evidence (scientific, contextual and colloquial) required to answer the question of evaluation?" The questions of efficacy, effectiveness, appropriateness and implementation will be subordinated to the latter.

As we have seen, the issues under consideration are often multidimensional, that is, they include scientific, political, juridical, professional, ethical, economic or organizational aspects. Therefore, both contextual evidence and colloquial evidence contribute, along with scientific evidence, to drawing the most reliable portrait of the variegated aspects of the problematic situation, as a basis for supporting decision-making and clinical practice. This is why, when scientific evidence is insufficient, missing or non-conclusive, it is still possible to produce recommendations based on the available contextual and colloquial evidence. The recommendations are the result of the mediation between the three types of evidence.

This mediation process reflects the organic nature of the healthcare system that has to take into account the interdependency and complementarities of its components. The relevance of introducing a new technology or procedure in clinical practice, for example, cannot be assessed without considering alternative options, the necessary professional training, clinical judgment, the organization of services and the available resources.

### What action?

In most knowledge-to-action models, action is defined as "the use of knowledge by practitioners, policymakers, patients, and the public" [20]. In other words, the main preoccupation is to ensure the application of research findings.

In the context of HTA practices, the action to be taken is conceived at the beginning of the process. The researchers will implicitly ask themselves the following: What kind of action is implied by the request? Is it a matter of introducing a new technology, for which specific training and regulation could be necessary? Are we talking about a problem of primary-care management? Or does it concern health policy?

Therefore, it is the nature of the action under consideration that shapes and gives sense to knowledge production. Action is the impulse as well as the result of the social interactions that will produce knowledge.

In healthcare systems, each action engages more than one actor and implies a shared understanding of it. Thus, discussions are necessary to unveil the motivations and specific knowledge of the actors in order for concerted action to be taken.

### What role for the actors?

Actions are necessarily carried out by actors. The implementation of measures to improve healthcare and services cannot be realized without the knowledge, know-how, support and collaboration of all concerned actors. As expressed by Lomas et al. [25] (p. 7), decision-makers are "sensitive to both scientific rationality and the local rationality of the workplace" for guidance.

In the literature on knowledge translation, we often find the terms "producers" and "users", which relate to the semantics of research and development. From this perspective, users are considered as clients that need to be convinced by producers of the benefits of the proposed research findings. This explains why, for some authors, the main challenge consists in tailoring knowledge in such a way that it will reach its target audience [10,21]. Lavis and his colleagues [10] have identified five essential questions which should be answered in order to render research results actionable, that is, applicable by the users: What should be transferred? To whom? By whom? How? With what effect?

Implicitly, the notion of users and producers tends to put the focus on knowledge dissemination and implementation, which represents the end of the process. It also separates the actors into these two categories, which do not take into account the complexity and variety of roles actors involved in the process may play. Moreover, it confronts producers (researchers) and users (practitioners, managers, decision-makers, etc.), placing them face to face. This separation between actors may create disequilibrium as regards to how their roles will be conceived.

The use of the term stakeholders seems more appropriate to reflect the political nature of AETMIS' context of practices. It implies that every actor concerned by the project, including researchers, should be involved, one way or the other, according to their role in the problem under study and the issues at stake.

How the knowledge-to-action process is conceptualized will have an impact on how the role of actors will be defined. Some models depict a unidirectional process, where knowledge is considered a product to transmit to users (knowledge push) [35]. Also falling into this category is the production of research in response to a user's demand or need (users push).

Representative of most models, according to Ward et al. [30], is a cyclical or bidirectional approach to knowledge, where users and producers interact throughout the knowledge-to-action process, from knowledge production to its implementation. Its objective is to integrate the preoccupations and contexts of the users in the research problem's definition, and in the interpretation, validation and diffusion of the results [36,37]. The interactions aim to help "producers" understand the context of "users" in order to identify barriers and facilitators as regards the use of research findings [7,20,21] and to determine which knowledge translation strategies to choose [8,23].

The projects we have observed at AETMIS reveal a multidirectional and dynamic approach to knowledge production. Knowledge comes from different sources and involves actors from various sectors of the health system [30], who play different roles in the issues addressed and the data collection process. Part of knowledge is thus construed through the rationalization of each other's standpoint (roles, attitudes, relationships, knowledge, know-how, expertise, experience, etc.). Stakeholders are considered as co-producers of knowledge.

### HTA: a knowledge mobilization process?

The practices we have observed revealed an approach to knowledge production, dissemination and implementation which was multidimensional, organic, multidirectional, dynamic, and dependent on interactions with stakeholders.

In this context, HTA could be considered as a knowledge mobilization process per se. We define knowledge mobilization as the gathering and mediation of knowledge coming from different sources and various actors as a prelude to concerted action. This concept implies putting knowledge into action or, more precisely, as Elissalde and Renaud [36] (p. 415) would define it, "all the types of knowledge, whether they come from research or practical experience". Mobilizing signifies preparing for action from the very beginning. For Phipps and Shapson [38], "knowledge mobilization encompasses methods of knowledge transfer, translation and exchange and extends them to include the co-production of knowledge. Knowledge mobilization turns research into action" (p. 213).

That is why, rather than knowledge to action, this process could be qualified as "knowledge in action." It is through the HTA process that the global portrait and the types of evidence to produce, exchange, integrate and interpret will appear. Moreover, it is through interactions and the co-construction of knowledge that the most appropriate interventions to recommend and the conditions of their applicability will emerge.

The nature of the projected action leads and shapes the whole process of knowledge production, dissemination and implementation. For instance, if the action concerns a safety problem, the HTA agent will have to mobilize the relevant knowledge, that is, evidence concerning the risks inherent in the technology or procedure under consideration, the qualifications required and the existing regulations framing their use, as well as the key actors concerned with each of these aspects and their practical experience and knowledge on the subject.

Defining HTA as a knowledge mobilization process could provide a conceptual basis to overcome a difficulty many authors have raised, i.e. the problem of integrating social, political and ethical issues in HTA [39,40], which, if lacking, would undermine the capacity of most HTA agencies to influence policy and practice appropriately and effectively [3,6,40,41]. Such a difficulty could be explained in part by the "absence of strong theoretical foundations" [42]. More precisely, the field of HTA has centered much of its effort on "strengthening its methodological foundations while rendering its purpose and epistemological basis largely undertheorized" ([43] (p. 197) in [1] (p. 1520))

Since at the beginning of the process there is a request stemming from a problematic situation, this implies that the ensuing assessment will be framed in social, political and ethical terms (What is the problem? What are the issues? Who is involved? What are the possible actions that can be undertaken?). The related issues, the types of evidence needed to address them, and the social actions its problem-solving will entail become the guiding elements of the knowledge production, dissemination and implementation process. As such, HTA agencies play the role of mediator between different orders of knowledge and the various actors concerned by the issues.

In other words, when HTA is conceived as a social process instead of a technical one, it opens the scope of analysis and justifies and supports the integration of social, political and ethical issues.

AETMIS HTA practices, context and mandate offer the conditions for such a mobilization of knowledge and actors, and their mediation, in view of supporting decision-making. Indeed, the Agency is neither a research institute, nor an association or interest group, nor does it make any decisions or provide healthcare or services. The Agency could be considered a boundary organization, "which functions as an intermediary agent to different principals: researchers, professionals, policy makers and/or citizens. By projecting the delegated authority from all principals involved, the boundary organization collects and integrates different resources, and thus is responsive to all of them" [36] (p. 239).

The fact that our research was not initially intended to draw a reflection on HTA in general, but to describe, in a short time and for internal purposes only, emerging practices of knowledge production and dissemination at a given HTA agency constitutes a limit. We have only examined a small sample of the agency's projects, which does not necessarily reflect all of the organization's practices. Moreover, our narrative literature review of knowledge translation models might have left aside models that could have applied to HTA practices. Nevertheless, our analysis has revealed the necessity for HTA to develop an approach to knowledge production and dissemination that takes into account the particularities of its practices context and of its mission as a support to decision-making.

## Conclusion

The objective of this exploratory case study was to examine the knowledge production, dissemination and implementation process in a HTA and clinical guidance context. The analysis of six projects conducted at AETMIS revealed a comprehensive approach to knowledge and interactions, which differs fundamentally from the characteristics of most dominant knowledge translation models in the healthcare sector.

Most knowledge translation models propose strategies to move evidence more effectively into practice and decision-making. Their starting point is the need for research findings to be disseminated and implemented. Contextual evidence and colloquial evidence, as well as interactions with stakeholders, are considered useful in identifying barriers and facilitators as regards the application of scientific evidence.

In the context of HTA, projects begin with a request raised in a problematic situation. Their purpose is to solve a problem by mobilizing all the types of evidence required and the actors concerned by the issues under consideration, in order to support political, organizational or clinical decision-making. Therefore, health technology assessment relies on the mediation between contextual, colloquial and scientific evidence, as well as on interactions with stakeholders for recommendation making.

From this perspective, the concept of knowledge mobilization seems more appropriate to describe the multidimensional, multidirectional and organic HTA approach observed in this study. Moreover, defining HTA as a knowledge mobilization process per se might contribute to consider the different orders of knowledge, the social, political and ethical dimensions, and the interactions with stakeholders, among the essential components required to respond to the preoccupations, needs and contexts of all actors concerned with the issues of the evaluation question under study.

This definition of HTA puts forward the utilization-focused nature of evaluation, as described by Patton [28], which implies to seek with the concerned and accountable stakeholders actionable answers for decision-making. Moreover, conceiving HTA as a knowledge mobilization process could offer a rational basis to justify from the beginning the public and patient involvement in health technology assessment and coverage decisions, which is becoming an increasing concern within HTA organizations and the object of current research [1,44-46].

Further investigation would be necessary to examine more systematically the implications and effectiveness of defining HTA as a knowledge mobilization process, for a more thorough consideration of ethical, social and political issues, as well as of a mean of involving stakeholders, patients and the public through the whole knowledge production and dissemination process.

## Competing interests

The author declares no competing interest.

## Authors' contributions

MF carried out the case study and drafted the manuscript. MF is the only author of this manuscript.

## Author's information

MF is a sociologist of science currently working as a consulting researcher at Quebec's *Institut national d'excellence en santé et en services sociaux *(INESSS), which succeeded to the *Agence d'évaluation des technologies et des modes d'intervention en santé et services sociaux *and the *Conseil du médicament*, in January 2011.
